# Endoscopic Retrieval of a Migrated Biliary Stent Causing Duodenal Perforation in a Patient With Mirizzi Syndrome

**DOI:** 10.7759/cureus.96339

**Published:** 2025-11-07

**Authors:** Mohammed Zaid Jaffar Hussain Desai, Yovan Sumaruth, Javaid Iqbal, Sajjad Mahmood, Venkata P K. Lekharaju, Mohammed Korani

**Affiliations:** 1 General Internal Medicine, Tameside and Glossop Integrated NHS Foundation Trust, Manchester, GBR; 2 Internal Medicine, North Manchester General Hospital, Manchester, GBR; 3 Gastroenterology, Manchester Foundation Hospitals, Manchester, GBR; 4 Gastroenterology and Hepatology, Wythenshawe Hospital, Manchester University NHS Foundation NHS Foundation Trust, Manchester, GBR; 5 Gastroenterology, Wythenshawe Hospital, Manchester, GBR; 6 Gastroenterology and Hepatology, North Manchester General Hospital, Manchester, GBR

**Keywords:** biliary stent, biliary stent migration, duodenal perforation, endoscopy ercp, non surgical retrieval

## Abstract

Preoperative endoscopic retrograde cholangiopancreatography (ERCP) with biliary stenting is a widely used intervention to achieve biliary drainage in patients with Mirizzi syndrome. While ERCP offers a minimally invasive approach to relieve obstruction and aid surgical management, it is not without risks. Reported complications of biliary stenting include cholangitis, pancreatitis, stent occlusion, and migration. In rare circumstances, migrated stents may result in intestinal perforation, most often involving the duodenum. Such events can be serious and require prompt recognition and management to prevent secondary complications such as peritonitis or intra-abdominal sepsis.

We describe the case of a woman in her twenties who developed a delayed duodenal perforation caused by the distal migration of a straight plastic biliary stent following ERCP for Mirizzi syndrome. The stent traversed the second part of the duodenum and extended into the retroperitoneal space down to the pelvis. It was successfully removed endoscopically under sedation, with defect closure achieved using endoscopic clips. This case highlights a rare but significant complication of biliary stenting and demonstrates the feasibility of safe endoscopic management.

## Introduction

Preoperative endoscopic retrograde cholangiopancreatography (ERCP) with biliary stenting is a key intervention for biliary drainage in patients with Mirizzi syndrome. ERCP is a minimally invasive option to relieve biliary obstruction and facilitate surgical management; it is not without risks. Complications of biliary stenting include cholangitis, pancreatitis, stent occlusion, and migration. Distal migration of plastic biliary stents is reported in 5-10% of cases. [1] Approximately 1% of migrated stents cause intestinal perforation, most commonly in the duodenum [2][3]. This can be a potentially serious event that needs early recognition and management to avoid progression of secondary complications such as peritonitis or intra-abdominal sepsis. 

We report a rare case of a woman in her 20s who developed a delayed duodenal perforation due to the distal migration of a straight plastic biliary stent following ERCP for Mirizzi syndrome. The stent perforated the second part of the duodenum and extended throughout the retroperitoneal space into the pelvis. This was successfully removed endoscopically under sedation with defect closure by endoscopic clipping. This case presents a rare complication of biliary stenting and the safety of its endoscopic management. 

## Case presentation

A woman in her 20s, scheduled for an elective cholecystectomy due to calculous cholecystitis, presented to the Accident and Emergency Department with escalating right upper quadrant and epigastric pain, bilious nausea and vomiting, and jaundice with pale stools.

Initial investigations

Liver function tests demonstrated a cholestatic-hepatitic pattern, with markedly elevated alanine aminotransferase (ALT), alkaline phosphatase (ALP), and bilirubin levels (Table 1). 

**Table 1:** Liver function tests.

Test	Patient value	Reference range
Alanine transaminase (ALT)	1,045 IU/L	0-35 IU/L
Alkaline phosphatase (ALP)	451 IU/L	30-130 IU/L
Total bilirubin	101 µmol/L	0-21 µmol/L
Albumin	46 g/L	35-50 g/L
Globulins	28 g/L	15-42 g/L
Total protein	74 g/L	60-80 g/L

Upon examination, she was vital and stable. A magnetic resonance cholangiopancreatography (MRCP) without contrast showed a 14-mm cystic ductal calculus, possibly causing compression on the common bile duct (CBD), suggestive of Mirizzi syndrome. 

**Figure 1:**
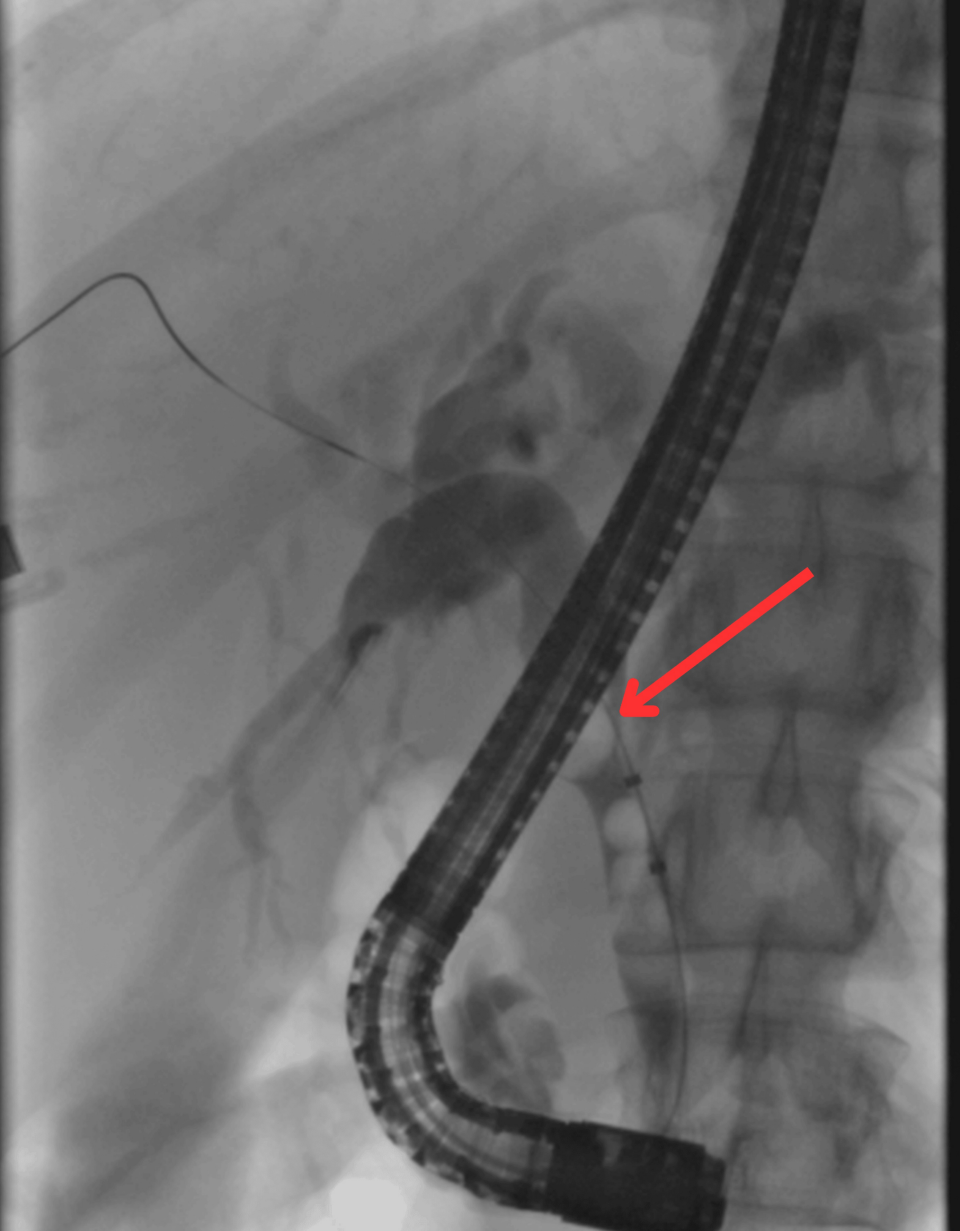
ERCP image showing a single, fixed filling defect in the cystic duct, consistent with a stone. ERCP, endoscopic retrograde cholangiopancreatography

The patient was referred for an ERCP before exploring surgical options. ERCP was performed, where a single significant fixed filling defect consistent with a stone was visualized in the cystic duct (Figure 1).

**Figure 2:**
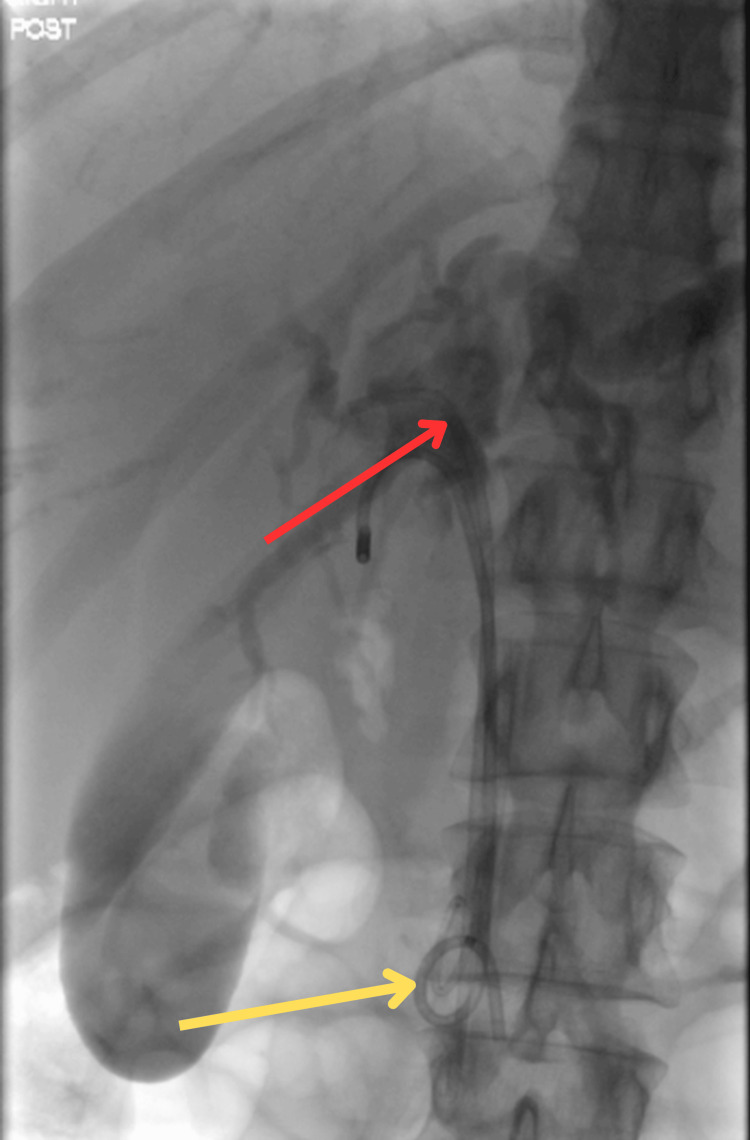
Fluoroscopic (C-arm) view during ERCP showing a single flanged straight plastic stent (red arrow) and a double-pigtail plastic stent (yellow arrow) successfully placed in the common bile duct.

One 7 Fr × 9 cm straight plastic stent and one 7 Fr × 7 cm double-pigtail plastic stent were successfully placed in the CBD to ensure optimal biliary drainage. A single flanged straight plastic stent was successfully placed in the pancreatic duct to reduce the risk of pancreatitis following inadvertent pancreatic duct cannulation (Figure 2).

Afterward, the patient underwent an elective cholecystectomy when the 7 Fr x 7 cm double pigtail plastic stent biliary stent was removed. Two weeks postoperatively and approximately eight weeks after the initial ERCP, she presented to the ED with severe abdominal pain, chest pain, and shortness of breath. On admission, the patient had an elevated D-dimer, obstructive jaundice. Blood investigations demonstrated an ongoing obstructive picture with raised bilirubin and inflammatory markers (Table 2). 

**Table 2:** Blood results.

Test	Patient value	Reference range
Alanine transaminase (ALT)	752 IU/L	0-35 IU/L
Alkaline phosphatase (ALP)	196 IU/L	30-130 IU/L
Total bilirubin	69 µmol/L	0-21 µmol/L
Albumin	34 g/L	35-50 g/L
Globulins	27 g/L	15-42 g/L
Total protein	27 g/L	60-80 g/L
C-reactive protein (CRP)	67 mg/L	<6 mg/L

**Figure 3:**
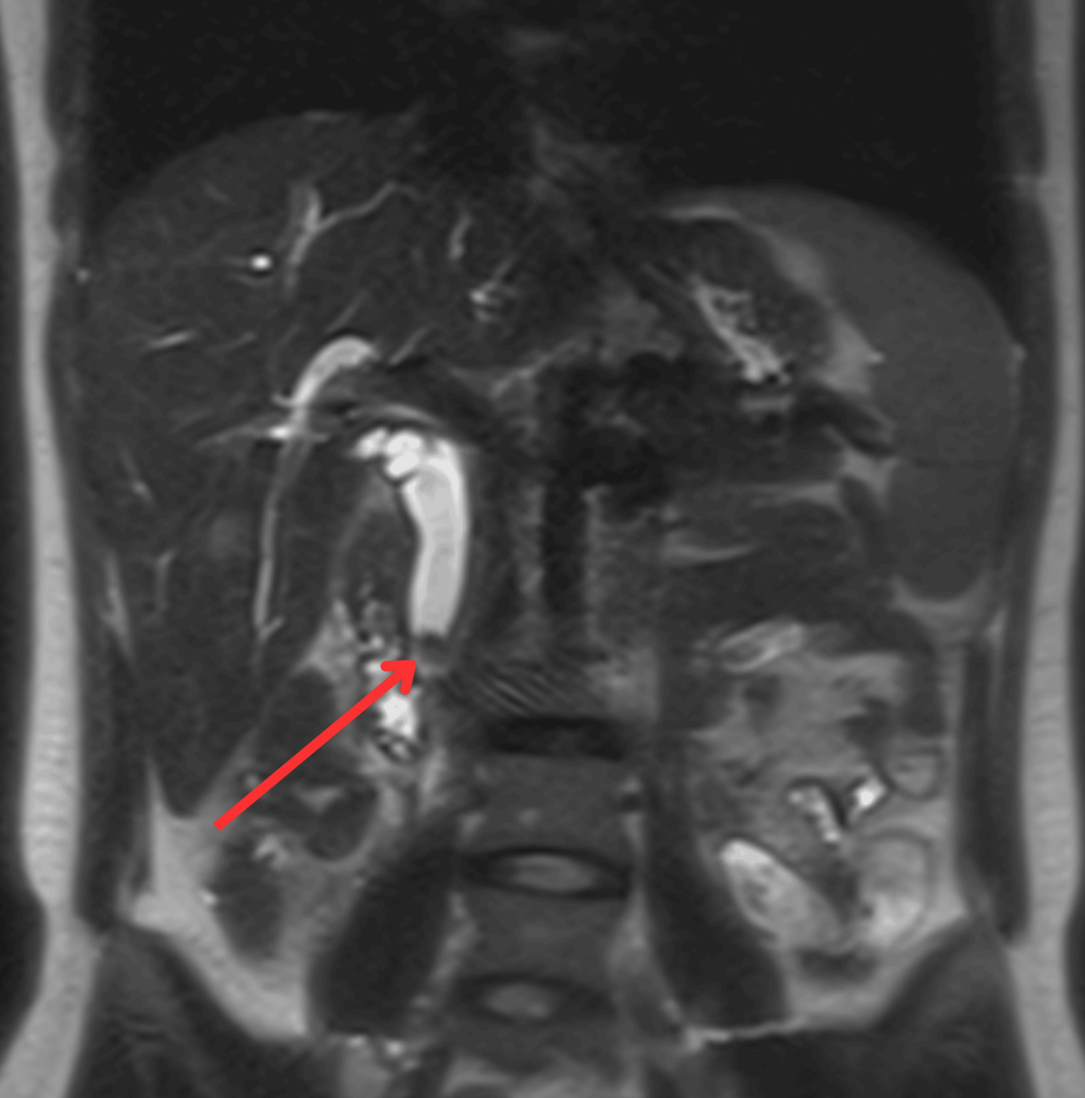
MRCP confirmed a 10-mm distal CBD calculus. MRCP, magnetic resonance cholangiopancreatography; CBD, common bile duct

She underwent a nuclear medicine (NM) lung perfusion scan, which ruled out pulmonary embolism. An abdominal and pelvic ultrasound revealed a potential stone in the distal CBD. An MRCP confirmed a 10 mm distal CBD calculus (Figure 3).

**Figure 4:**
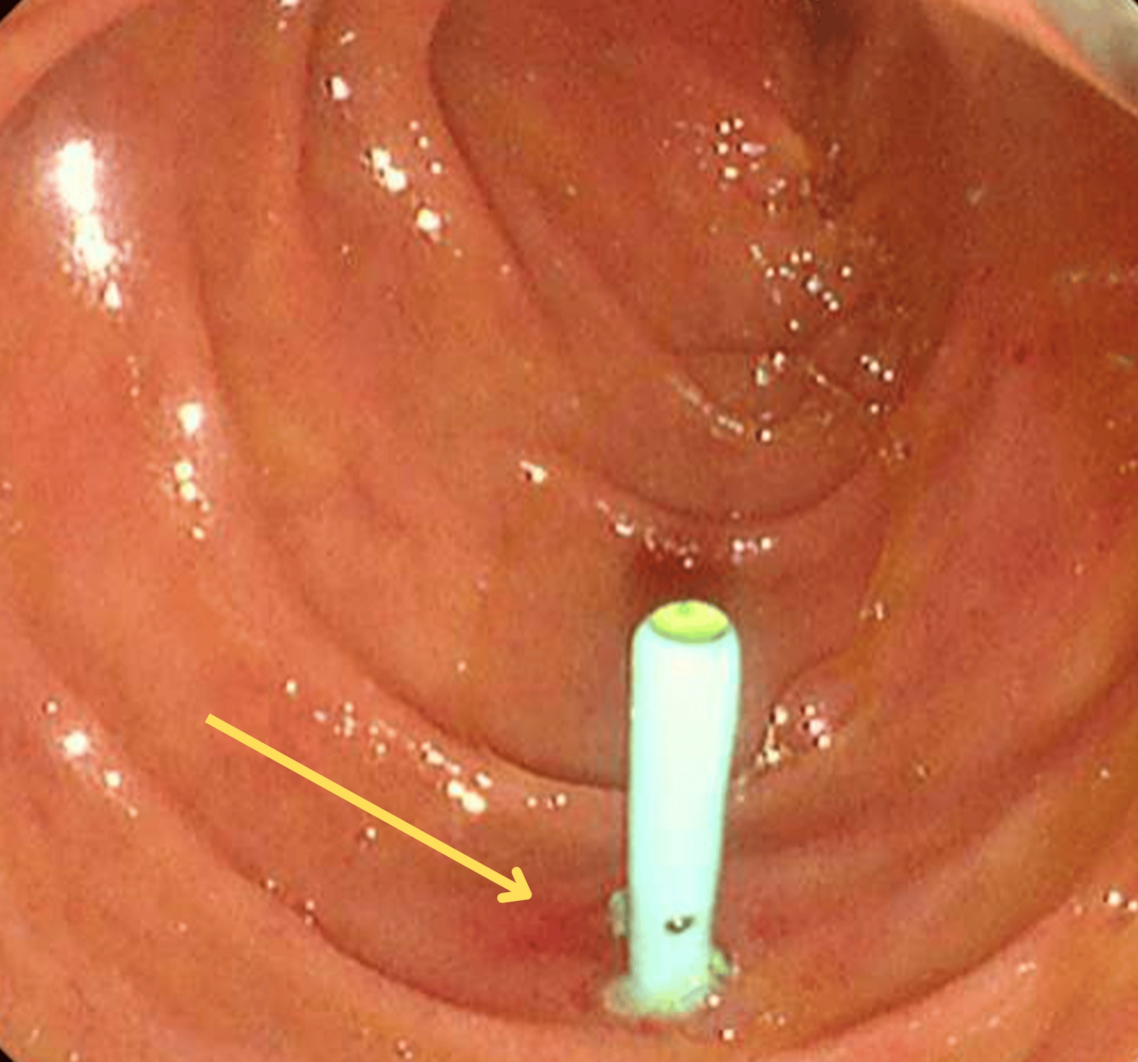
ERCP image showing a straight plastic stent on the opposite wall of the ampulla, migrating through the duodenal wall and suggesting perforation. ERCP, endoscopic retrograde cholangiopancreatography

On the second ERCP, the straight plastic stent was observed on the opposite wall of the ampulla, migrating through the duodenal wall and suggesting perforation (Figure 4).

**Figure 5:**
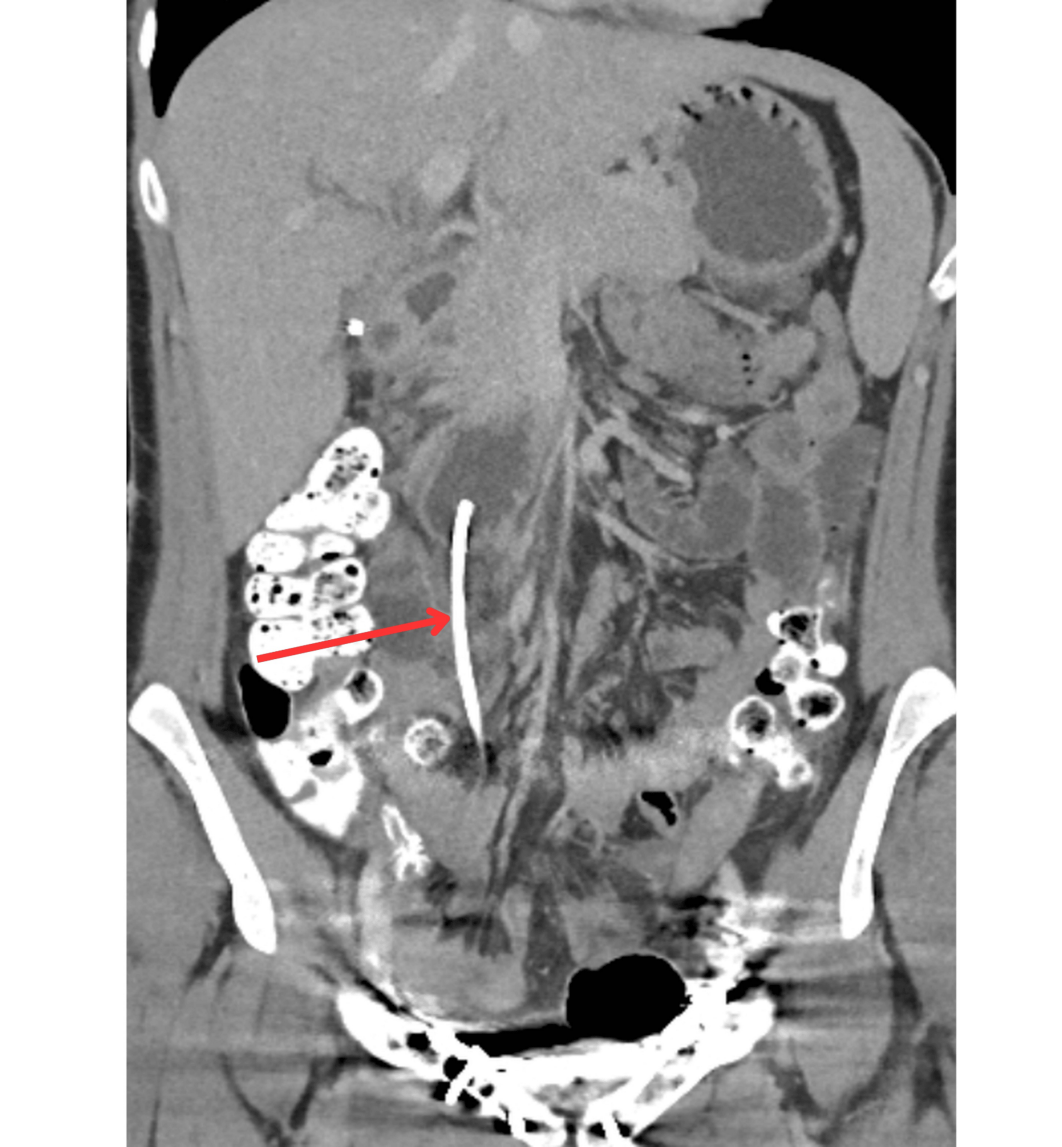
CT of the abdomen and pelvis showing approximately 17 mm of the stent lying within the lumen of the duodenum. The remainder of the stent protruded through the duodenal wall (approximately 10 cm long).

**Figure 6:**
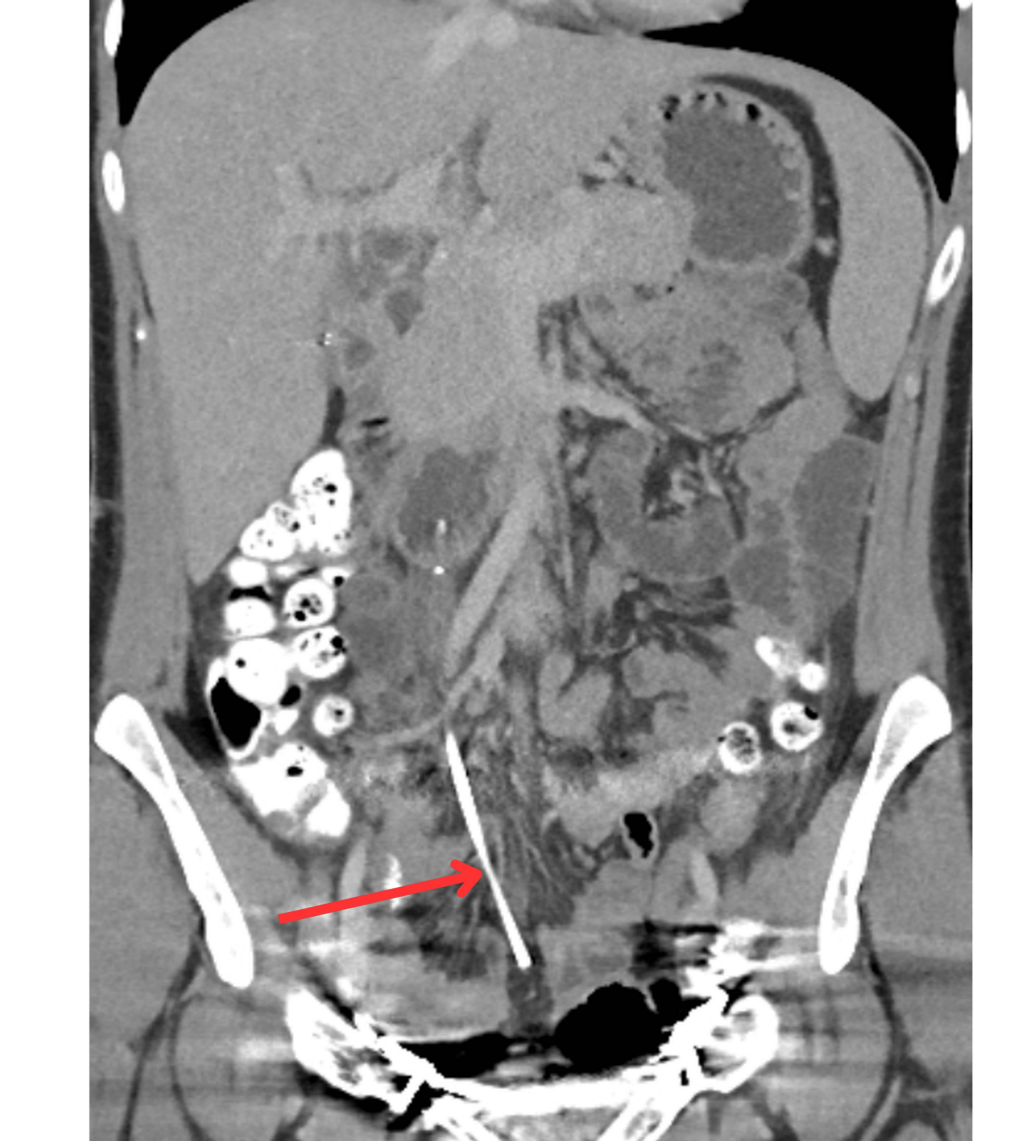
CT of the abdomen and pelvis showing the tip lying in the inferior aspect of the small bowel mesentery near the expected level of the urinary bladder.

The plan was to proceed with ERCP, where duct clearance was achieved, and arrange a computed tomography (CT) abdomen and pelvis scan to assess the migrated stent, which reported approximately 17 mm of the stent lay in the lumen of the duodenum. The remainder of the stent protruded through the duodenal wall (approximately 10 cm in length), with the tip lying in the inferior aspect of the small bowel mesentery near the expected level of the urinary bladder. No significant hemorrhagic complications or evidence of surrounding free gas were observed (Figures 5-6).

**Figure 7:**
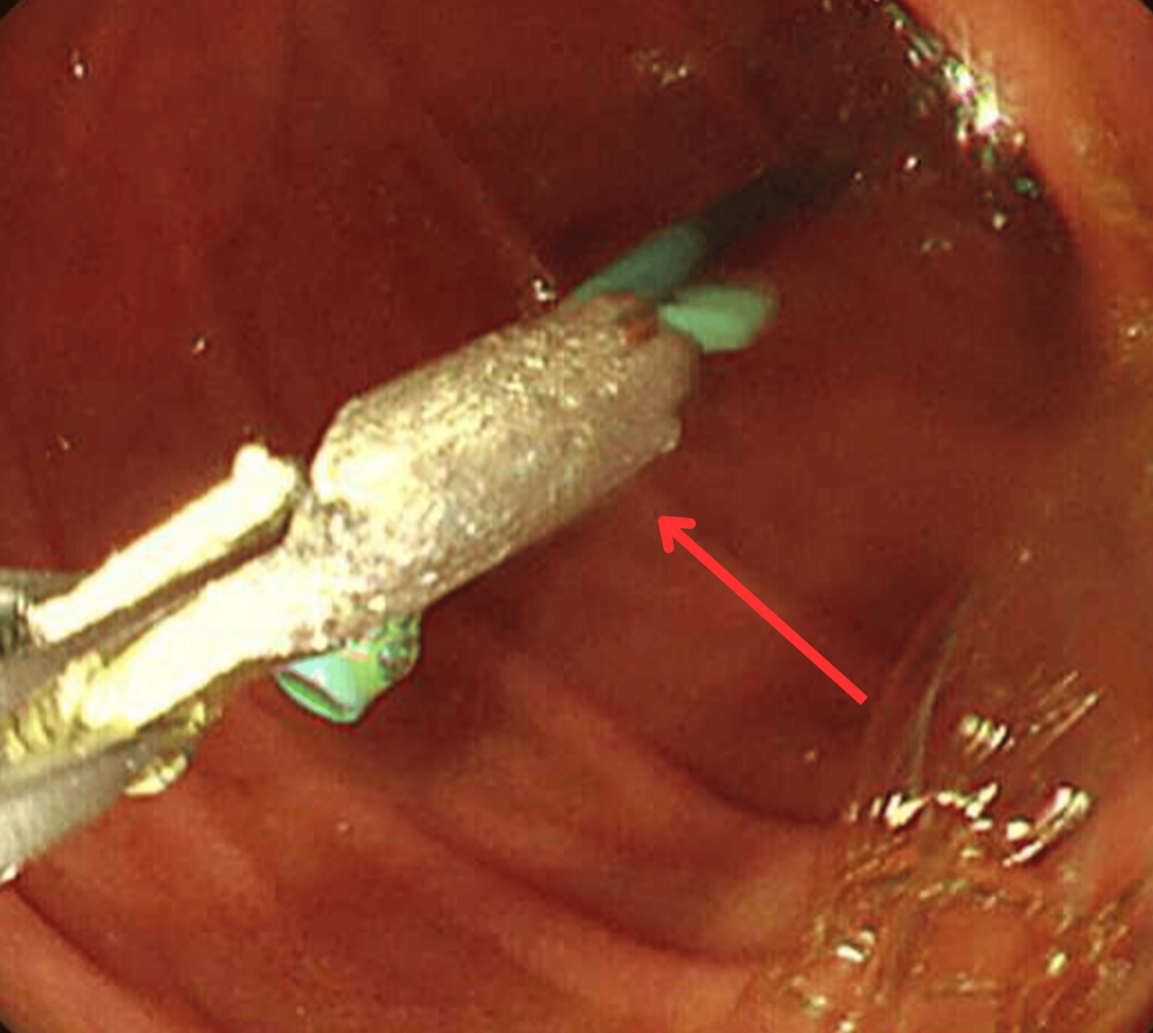
Endoscopic view of the stent being removed from the duodenal wall using biopsy forceps.

The case was discussed with the surgical team, and a plan was decided to proceed with endoscopic removal via forward viewing gastroscopy under fluoroscopy with sedation. The stent was removed using biopsy forceps from the duodenal wall (Figure 7).

**Figure 8:**
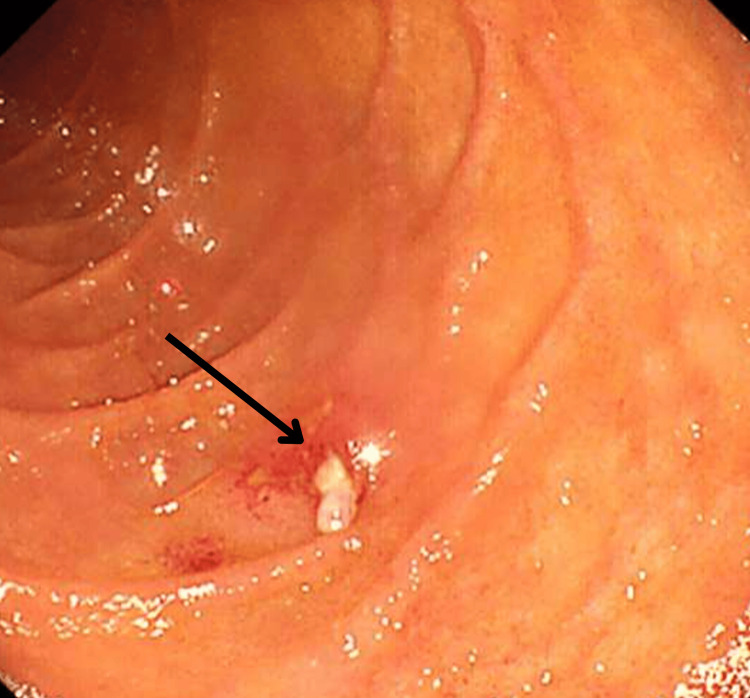
Endoscopic view during subsequent mucosal assessment confirming a small defect.

**Figure 9:**
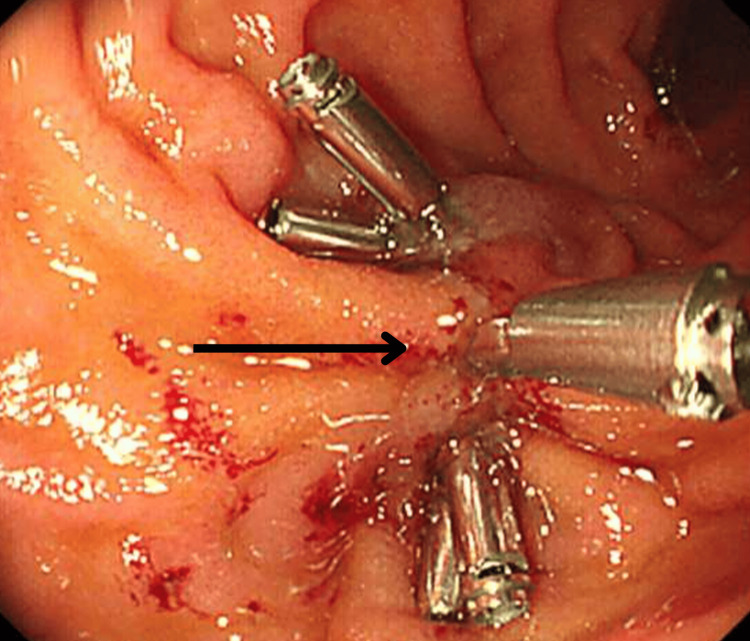
Endoscopic view showing five endoscopic hemoclips used to close the defect.

**Figure 10:**
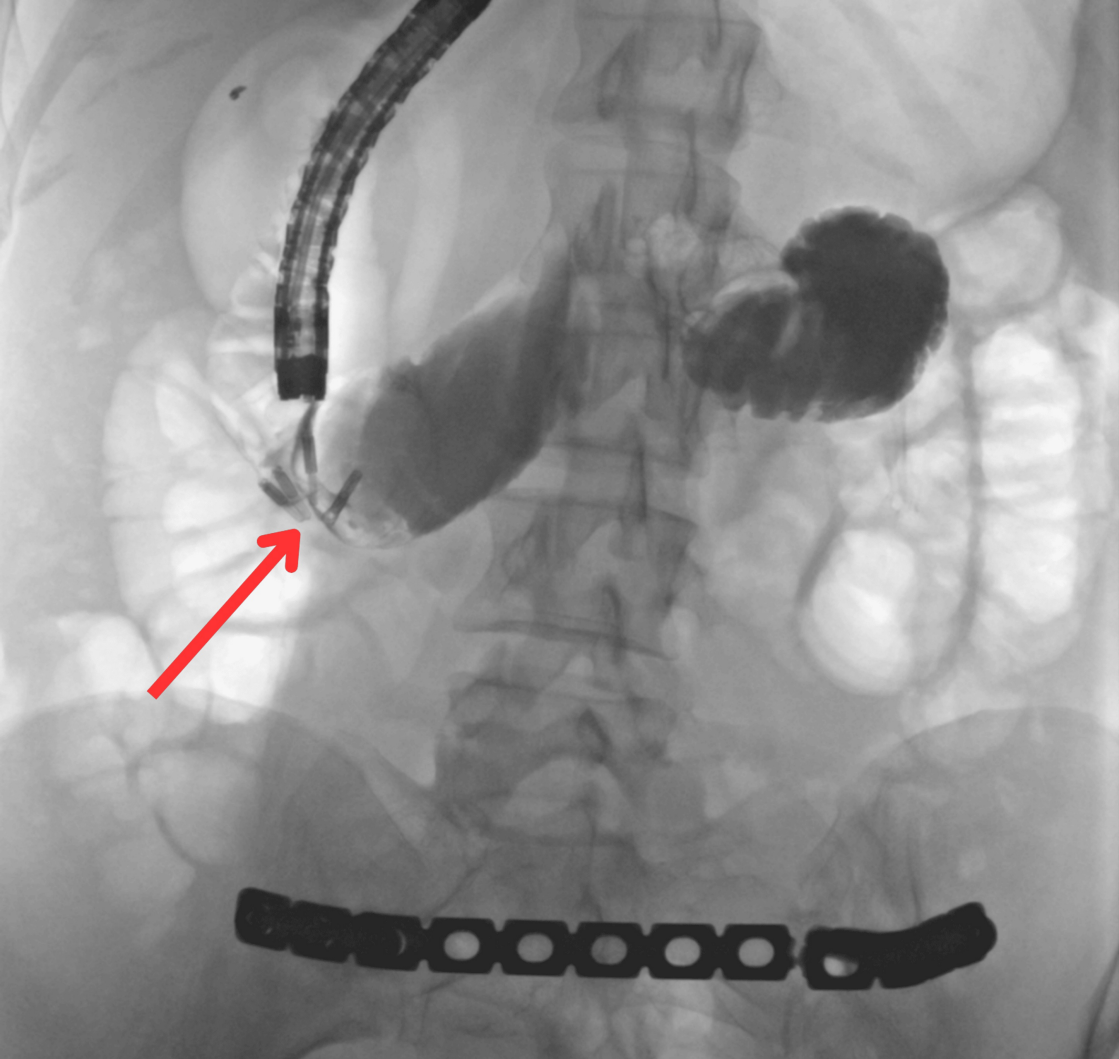
Fluoroscopic (C-arm) view following contrast injection, confirming no leakage after closure of the duodenal defect.

Subsequent mucosal inspection revealed a small duodenal defect (Figure 8), which was successfully closed with five endoscopic hemoclips (Figure 9). Contrast injection confirmed no leakage (Figure 10).

After a short period of observation, the patient was discharged home the same day.

## Discussion

ERCP with biliary stenting is the first-line approach in the management of biliary obstruction in both benign and malignant diseases, including Mirizzi syndrome. While stent-related complications such as occlusion, cholangitis, and pancreatitis are well recognized, distal migration with duodenal perforation remains a rare, significant event [4].

Straight plastic stents are more prone to migration compared to pigtail, and pigtail ends reduce migration risk by anchoring better [3], with reported migration rates ranging from 5% to 14% [3,5]. Duodenal perforation from migrated biliary stents occurs in less than 1% of these cases and is typically associated with prolonged time of more than four to six weeks, dilated bile duct of more than 10 mm, and the use of larger-caliber or straight stents [6].

In our case, the patient had undergone ERCP with placement of both straight and pigtail plastic biliary stents for decompression of the biliary tree. Although one stent was removed during her cholecystectomy, a second stent was later found to have migrated distally, perforating the second part of the duodenum and extending into the pelvic cavity. CT is widely utilized as the primary modality to assess and evaluate any stent-related complications [2]. The delayed presentation of our patient, two weeks post-surgery and six weeks post-index ERCP, highlights the importance of maintaining a high index of suspicion for stent-related complications even after initial clinical improvement.

Recent literature identifies several key risk factors for distal biliary stent migration: benign biliary strictures, a dilated bile duct >10 mm, straight stent design, use of a single stent, and prior sphincterotomy. Time-related risks such as delayed stent removal are also significant, with a median time to perforation of 44.5 days reported in a recent systematic review [6].

Surgical repair has traditionally been the standard approach, with 42% endoscopic success and 17% overall mortality for gastrointestinal perforations, particularly when complicated by peritonitis in unstable patients [6]. A recent case series and reports suggest that endoscopic treatment may be effective in small, contained perforations without systemic signs of sepsis [7,8]. Endoscopic management was chosen in our case after discussion with the surgical team, as the patient was stable with no clinical and radiological evidence of secondary complications from the migrated stent. The stent was successfully removed endoscopically using forceps via gastroscopy, and the mucosal defect was closed with endoscopic clips. The patient was discharged the same day and remained well at the seven-day follow-up. This case helps us to understand the feasibility and safety of minimally invasive management in appropriately selected patients. However, large-scale studies and randomized controlled trials are needed to guide definitive management. Individual decisions are often based on the patient’s stability, perforation size, location, available expertise, and available resources [9].

In our case, the use of endoscopic clips achieved secure closure, which is the most common method used, as well as providing a good outcome [7]. Further leakage was ruled out with intraluminal contrast under fluoroscopy. This case confirms that endoscopic therapy can be safely used as a first-line approach in selected patients with small duodenal perforations.

## Conclusions

This case highlights a rare but potentially serious complication of biliary stenting-duodenal perforation secondary to stent migration. Our case demonstrates that endoscopic intervention, when performed promptly and in centers with appropriate expertise, can provide a safe and effective alternative to surgical management, resulting in reduced recovery time and avoidance of major operative risks.

Importantly, this case emphasizes the need for heightened clinical vigilance in patients presenting with abdominal pain or obstructive symptoms after biliary stent placement, as early imaging and endoscopic evaluation can be critical for diagnosis. It also underscores the importance of considering stent migration as a differential diagnosis in post-ERCP patients who re-present with unexplained symptoms.
